# Non-targeted metabolomics and network pharmacology of Taohong Siwu Decoction in hepatic fibrosis mouse model using high resolution mass spectrometry

**DOI:** 10.3389/fmolb.2025.1614341

**Published:** 2025-06-30

**Authors:** Shengsheng Li, Shenglan Qi, Rongsheng Li, Fangming Yang, Zhenyi Niu, Wei Liu, Suping Ma, Zhun Xiao

**Affiliations:** ^1^ Department of Digestive Diseases, The First Affiliated Hospital of Henan University of Chinese Medicine, Zhengzhou, China; ^2^ The First Clinical Medical College, Henan University of Chinese Medicine, Zhengzhou, China; ^3^ Shanghai Key Laboratory of Traditional Chinese Medicine, Shanghai University of Traditional Chinese Medicine, Shanghai, China; ^4^ Department of Pathology, The First Affiliated Hospital of Henan University of Chinese Medicine, Zhengzhou, China

**Keywords:** hepatic fibrosis, non-targeted metabolomics, molecular docking, Taohong Siwu decoction, mice, high resolution mass spectrometry

## Abstract

**Introduction:**

Taohong Siwu Decoction (THSW Decoction), a classic formula for treating blood stasis, has demonstrated significant clinical efficacy in the treatment of hepatic fibrosis. However, its primary active components and mechanisms of action remain unclear.

**Methods:**

In this study, a carbon tetrachloride (CCl_4_)-induced hepatic fibrosis mouse model was established to evaluate THSW Decoction’s therapeutic effects. Ultra-high performance liquid chromatography-quadrupole/Orbitrap high-resolution mass spectrometry (UHPLC-Q-Exactive Orbitrap HRMS) was employed to identify the main prototype chemical components of THSW Decoction in the blood, while non-targeted metabolomics analysis was performed using a Waters Synapt G2-Si QTOF mass spectrometer (Synapt G2-Si QTOF HRMS system). Network pharmacology and metabolomic data were integrated to elucidate the therapeutic targets, differential metabolites, and signaling pathways of THSW Decoction. Molecular docking and binding affinity predictions between key targets and principal compounds were performed using PyMOL software. Furthermore, molecular dynamics simulations were conducted to evaluate the structural stability and binding interactions. Finally, core targets were validated *in vivo*.

**Results:**

THSW Decoction effectively reduced CCl_4_-induced serum alanine aminotransferase (ALT) and aspartate aminotransferase (AST) levels, alleviating liver inflammation and collagen deposition. Forty-five blood components were detected, with 616 corresponding drug targets identified, including 419 overlapping targets for anti-hepatic fibrosis. The core protein-protein interaction (PPI) network comprised 59 nodes and 570 edges. Enrichment analysis revealed that THSW Decoction’s blood components primarily modulated biological processes such as positive regulation of response to external stimuli and oxygen content. Key signaling pathways included PI3K-Akt, estrogen, relaxin, and MAPK. Non-targeted metabolomics identified 148 differential metabolites between the model and normal groups, and 156 between the THSW Decoction and model groups. Thirty-five overlapping metabolites were enriched in cAMP, phospholipase D, and GnRH signaling pathways. Twenty intersection targets linked blood components, metabolites, and hepatic fibrosis. PPI analysis ranked JUN, PTGS2, BCL2, ESR1, and PPARG as the top five targets. A “drug-component-target-metabolite” network highlighted ferulic acid, p-hydroxycinnamic acid, 3-hydroxy-4-methoxycinnamic acid, ferulaldehyde, and vanillic acid as the top five blood components. Molecular docking and molecular dynamics simulations revealed that 3-hydroxy-4-methoxycinnamic acid binds stably to the core target PPARG, exhibiting a binding free energy of −93.68 kJ/mol. *In vivo* validation showed that THSW Decoction upregulated JUN and downregulated ESR1 expression in the liver.

**Discussion:**

This study elucidates THSW Decoction’s key blood components, potential targets, and mechanisms in the treatment of hepatic fibrosis, providing a foundation for further research.

## 1 Introduction

Liver fibrosis is a dynamic pathological process characterized by excessive deposition of extracellular matrix (ECM), primarily driven by chronic liver injuries such as viral hepatitis, alcoholism, nonalcoholic fatty liver disease (NAFLD), or autoimmune disorders (Roehlen et al., 2020). The activation of hepatic stellate cells (HSCs) is a central event in liver fibrogenesis. However, beyond HSCs, multiple cell types—including hepatocytes, liver sinusoidal endothelial cells (LSECs), and macrophages—are also involved in the initiation and progression of liver fibrosis. Current studies suggest that liver fibrosis is not the result of a single-cell action, but rather the outcome of complex intercellular interactions. For example, hepatocyte injury and death (Roehlen et al., 2020), LSEC dedifferentiation (Gao et al., 2024), and pro-inflammatory polarization of macrophages (Matsuda and Seki, 2020) can all induce HSC activation and proliferation. In turn, activated HSCs can exacerbate hepatocyte injury, LSEC dedifferentiation, and macrophage pro-inflammatory polarization, thereby creating a vicious cycle that perpetuates fibrogenesis (Akkiz et al., 2024).

Without intervention, this condition progresses to cirrhosis, severely endangering patients’ lives. Studies indicate the annual number of cirrhosis-related deaths rose from 899,000 in 1990 to 1.32 million (GBD, 2017 Cirrhosis Collaborators, 2020). Therefore, early intervention to reverse or delay fibrosis progression helps reduce rates of decompensated cirrhosis and liver cancer, improve patients’ quality of life, and enhance disease prognosis (Ginès et al., 2022). Numerous studies show that etiological treatment (e.g., controlling or curing viral infections) can reverse hepatic fibrosis (Caligiuri et al., 2021). However, without targeted anti-fibrotic therapy, the disease frequently progresses (Schuppan et al., 2018). While many investigational drugs have undergone preclinical and clinical trials, no anti-fibrotic drugs have yet received Food and Drug Administration approval (Yoon et al., 2016).

Taohong Siwu (THSW) Decoction is a classic Chinese herbal formula in traditional Chinese medicine, composed of peach kernel, safflower, *Ligusticum wallichii*, Radix Rehmanniae Praeparata, Radix Paeoniae Alba, and Radix Angelicae Sinensis. THSW Decoction is primarily used to treat diseases caused by blood stasis. The pathogenesis of blood stasis is considered to be closely related to hepatic fibrosis formation (Wang et al., 2018). Recent studies have shown that THSW Decoction can alleviate liver fibrosis by improving hepatocellular lipid metabolism and mitochondrial autophagy (Wu et al., 2024). Our previous research further demonstrated that THSW Decoction significantly attenuates liver tissue damage and collagen deposition in fibrotic mice by regulating glutathione metabolism and inhibiting ferroptosis in hepatocytes (Xiao et al., 2025). However, as a multi-component traditional Chinese medicine formula, it remains unclear how the bioactive components absorbed into the bloodstream synergistically regulate glutathione metabolism and pathological processes beyond ferroptosis through multiple targets and signaling pathways. Moreover, whether THSW Decoction exerts its holistic therapeutic effects by modulating the endogenous metabolic network also warrants further investigation. Network pharmacology and non-targeted metabolomics have been widely used to investigate the components and mechanisms of traditional Chinese medicine. In this study, a CCl_4_-induced hepatic fibrosis mouse model was established to evaluate its efficacy. Ultra-high performance liquid chromatography-quadrupole/Orbitrap high-resolution mass spectrometry (UHPLC-Q-Exactive Orbitrap HRMS) technology was employed to analyze the prototype active components of THSW Decoction in blood. Network pharmacology, non-targeted metabolomics, molecular docking and molecular dynamics simulation were performed with a Waters Synapt G2-Si QTOF mass spectrometer (Synapt G2-Si QTOF HRMS) to explore its potential therapeutic mechanisms against hepatic fibrosis and validate these findings *in vivo*, providing a foundation for further research.

## 2 Materials and methods

### 2.1 Animal source

Thirty-two 6-week-old SPF-grade male C57BL/6N mice, weighing 16–18 g, were purchased from Beijing Vital River Laboratory Animal Technology Co., Ltd. (Beijing, China certificate number: 110011231102606376). The animals were housed in the Animal Experiment Center of Henan University of Chinese Medicine, with a room temperature of 25°C ± 2°C, relative humidity of 40%–60%, and a 12-h light-dark cycle, with free access to food and water. Animal experiments were approved by the Animal Welfare and Ethics Committee of Henan University of Chinese Medicine (approval number: IACUC-20230309-02).

### 2.2 Drugs and reagents

The experimental drugs were in granular form. Peach kernel was purchased from Guangdong Yifang Pharmaceutical Co., Ltd. (Foshan, Guangdong Province, China; batch number: 0111212); safflower, rehmannia glutinosa, white peony, and angelica were purchased from Jiangyin Tianjiang Pharmaceutical Co., Ltd. (Jiangyin, Jiangsu Province, China; batch numbers: 21016504, 21036474, 21026264, 21046824); *Ligusticum wallichii* was purchased from Beijing Kangrentang Pharmaceutical Co., Ltd. (Beijing, China; batch number: 20042411). THSW Decoction was prepared by mixing peach kernel, safflower, rehmannia glutinosa, white peony, angelica, and *Ligusticum wallichii* granules in a crude drug ratio of 3:2:4:3:3:2. Chromatographic-grade methanol, acetonitrile, and formic acid were all purchased from Sinopharm Chemical Reagent Co., Ltd. (Shanghai, China; batch numbers: 10014108, 40064160, 40023786).

### 2.3 Establishment of hepatic fibrosis model and Drug Administration

Thirty-two mice were randomly divided into four groups: the blank control group (C), CCl_4_-induced model group (CCl_4_), THSW Decoction low-dose group (L), and THSW Decoction high-dose group (H). The blank control group was administered by intraperitoneal injections of olive oil at a dose of 2 mL/kg body weight three times per week for 6 weeks. The hepatic fibrosis model was established by intraperitoneal injection of a 15% CCl_4_-olive oil solution at a dose of 2 mL/kg body weight three times per week for 6 weeks. Starting from the 4th week, the THSW Decoction groups received granular agents (2.2 or 4.4 g/kg body weight) via oral gavage, while the CCl_4_ group and blank control group were administered deionized water by the same method. After 6 weeks, the mice were anesthetized with pentobarbital sodium, and blood samples were collected from the inferior vena cava along with liver tissue specimens.

### 2.4 Detection of serum aminotransferases

Serum ALT and AST activities were measured using commercial kits (Nanjing Jiancheng Bioengineering Institute, Nanjing, Jiangsu Province, China; catalog numbers C009-2-1 and C010-2-1).

### 2.5 Histopathology and immunohistochemistry

Liver tissues were fixed in neutral formalin, dehydrated with ethanol gradient, transparent with xylene, embedded in paraffin, and cut into 4 μm thick sections. Hematoxylin-eosin (H&E) staining was used to observe the inflammatory damage and Sirius red (SR) staining was used to observe the collagen deposition. The expression of Col-Ⅰ in liver tissue was detected by immunohistochemistry (1: 1,000; Servicebio, Wuhan, Hubei Province, China; catalog number: GB11022-3).

### 2.6 Western blot analysis

Liver tissue was lysed in RIPA buffer containing a protease inhibitor cocktail and homogenized on ice. Protein concentration was determined using the bicinchoninic acid (BCA) assay. Protein samples (25–50 μg) were denatured at 100°C for 10 min, separated by 10% SDS-PAGE, and transferred to polyvinylidene fluoride (PVDF) membranes. The membranes were incubated overnight at 4°C with primary antibodies against C-JUN, ESR1, and ACTIN (all at 1:1,000; Servicebio, Wuhan, Hubei Province, China; catalog numbers: GB111604, GB111843, and GB11001, respectively), followed by incubation with HRP-conjugated goat anti-rabbit IgG secondary antibody (1:3,000; Servicebio, Wuhan, Hubei Province, China; catalog number: GB23303).

### 2.7 Preparation of drug-containing serum

Mice were fasted for 12 h before administration (with free access to water) and divided into a blank group (3 mice) and a THSW Decoction group (6 mice). The blank group received deionized water, while the THSW Decoction group received THSW Decoction by gavage at 10 mL/kg (equivalent to 15 g/kg of crude drug, ∼2× the human equivalent dose). At 1 h and 3 h post-administration, blood was collected from 3 mice in the THSW Decoction group by ocular enucleation. Serum was separated by centrifugation (8,000 rpm, 15 min, 4°C), and pooled blank serum, 1-h drug-containing serum, and 3-h drug-containing serum were prepared. Serum samples (200 μL) were protein-precipitated with 1 mL methanol, centrifuged (12,000 rpm, 10 min, 4°C), and the supernatant was dried under nitrogen at 37°C, reconstituted in 80 μL of 20% methanol, and centrifuged again for analysis.

### 2.8 Non-targeted metabolomics analysis of liver tissue

#### 2.8.1 Chromatographic conditions

Chromatographic separation was performed on a Waters ACQUITY UPLC system (Waters Corporation, Milford, MA, United States) equipped with an ACQUITY HSS PFP column (100 mm × 2.1 mm, 1.8 μm). The mobile phase consisted of 0.1% formic acid in water (solvent A) and 0.1% formic acid in acetonitrile (solvent B). The following gradient elution program was applied: 2%–40% B from 0 to 5.0 min, 40%–98% B from 5.0 to 17.0 min, held at 98% B from 17.0 to 20.0 min, and returned to 2% B from 20.1 to 21.0 min. The column temperature was maintained at 40°C, with a flow rate of 0.3 mL/min. The injection volume was set to 1.0 μL, and the total run time was 21 min.

#### 2.8.2 Mass spectrometry conditions

Mass spectrometric detection was conducted on a Waters Synapt G2-Si QTOF mass spectrometer (Waters Corporation, Milford, MA, United States) in both positive (ESI^+^) and negative (ESI^−^) electrospray ionization modes, scanning m/z 100–1,000. Capillary voltages were set at 3.0 kV (ESI^+^) and −2.5 kV (ESI^−^). Cone voltage, desolvation temperature (350°C), and gas flows (desolvation 600 L/h, nebulizer 50 L/h) were optimized per manufacturer’s instructions. Mass calibration was performed weekly or after maintenance using sodium formate clusters (Waters QTOF Calibration Kit), covering ions from m/z 69 to 1,350 (ESI^+^) and m/z 113 to 1,023 (ESI^−^), achieving ±2 ppm accuracy. Daily mass correction employed leucine enkephalin (1 μg/mL in 50% acetonitrile with 0.1% formic acid) as lock mass, with reference ions at m/z 556.2771 (ESI^+^) and 554.2615 (ESI^−^) to ensure ongoing mass accuracy.

### 2.9 Detection of blood components of THSW decoction

The UHPLC-Q Exactive HRMS procedure for the analysis of the blood components of THSW Decoction was performed according to a published protocol (Xiao et al., 2025).

Data acquisition and analysis were performed using Xcalibur 3.0 software. Molecular formulas were deduced based on precursor ions ([M + H]^+^, [M-H]^-^) and adduct ions ([M + Na]^+^, [M + HCOOH]^-^), with a mass error tolerance of ±10 ppm. Identification was achieved by comparing experimental data with reference standards, literature, and MS^2^ fragments from the MassBank database.

### 2.10 Acquisition of targets of components migrating to blood

The SMILE of the components was obtained through PuBChem, and then the relevant targets were obtained using the SEA (https://sea.bkslab.org/) database and the Tcsmp database (https://tcmspw.com/tcmsp.php). Oral bioavailability (OB) and drug-likeness (DL) were set to 30% and 0.18 (Ru et al., 2014), respectively. Finally, the targets were normalised by the Uniprot database (https://www.uniprot.org).

### 2.11 Acquisition of hepatic fibrosis targets

The search term “hepatic fibrosis” was uesd to Drugbank (https://www.drugbank.ca/), GeneCards (https://www.genecards.org/), TTD (http://db.idrblab.net/ttd/), OMIM (https://www.omim.org/), PharmGkb (https://www.pharmgkb.org/), and DisGeNET (https://www.disgenet.org) databases to obtain disease targets. The obtained targets were converted to standard gene names in UniProt database after removing duplicates. Potential anti-hepatic fibrosis targets of components migrating to blood were obtained based on components targets and disease targets. The bioinformatics platform (http://www.bioinformatics.com.cn/) was applied to visualize the results by outputting Venn diagram.

### 2.12 Screening of therapeutic targets

Venn diagrams were generated using the Bioinformatics Platform (http://www.bioinformatics.com.cn/) to identify overlapping targets between THSW Decoction blood components and hepatic fibrosis.

### 2.13 PPI network construction

Intersection targets were uploaded to STRING platform (https://string-db.org/) with the condition of “*HOMO sapiens*” and “confidence level ≥0.9”, and isolated nodes were excluded. Core targets were selected based on topological parameters (Betweenness, Closeness, Degree, Eigenvector, LAC) using Cytoscape 3.8.0.

### 2.14 GO and KEGG enrichment analysis

Functional enrichment analysis was performed using the DAVID database (https://davidbioinformatics.nih.gov/) for Gene Ontology (GO: biological processes, cellular components, molecular functions) and Kyoto Encyclopedia of Genes and Genomes (KEGG) pathways. Results were visualized as bubble plots.

### 2.15 Non-targeted liver metabolomics

Liver tissue samples were processed by Meiji Biotech (Shanghai, China), Detailed methods can be found in the Supplementary Material.

### 2.16 “Drug-component-target-metabolite” network construction

The chemical structures of the common differential metabolites were retrieved from the PubChem database, and potential targets were predicted using the SwissTargetPrediction platform. Only targets with a probability score greater than 0 were included (Daina et al., 2019). Intersection targets between blood components and hepatic fibrosis targets were subjected to protein-protein interaction (PPI) analysis. A comprehensive network was subsequently constructed in Cytoscape to identify key bioactive components and metabolites.

### 2.17 Molecular docking

The protein structure of the targets were obtained from the PDB database (https://www.rcsb.org/), and the nuclear MOL2 files of the components were obtained from the PubChem (https://pubchem.ncbi.nlm.nih.gov). The ligand and receptor of the target protein were screened by the Pymol software. Molecular docking was performed using AutoDockTools 1.5.6 and AutoDockVina software, and the results were visualized by the Pymol software.

### 2.18 Molecular dynamics simulation

Molecular dynamics (MD) simulations were performed on the top-ranked target (with the highest binding free energy) and its corresponding blood-entering compound identified in Section 2.17. Simulations were conducted using the Amber 24 software package to evaluate the binding stability of the native protein and its mutant in complex with the same ligand (Case et al., 2005). System construction was carried out using the LEaP module by loading the PDB structures of both the wild-type and mutant proteins. The ff14SB force field was applied to describe the protein (Maier et al., 2015), while the General Amber Force Field (GAFF) was used to parameterize the ligand (Wang et al., 2004). Each system was solvated in a TIP3P water box with a 10.0 Å buffer from the protein surface, and neutralized by adding Na^+^ and Cl^−^ ions (Zulfat et al., 2024).

To eliminate steric clashes and bad contacts in the initial structures, a two-step energy minimization was performed: first, the solvent and ions were minimized with restraints on the protein backbone, followed by full minimization of the entire system without restraints using the steepest descent and conjugate gradient methods to ensure system stability.

The equilibration phase included two steps. In the first step, the system was gradually heated to 300.0 K under constant temperature (NVT) conditions with restraints on the protein backbone. In the second step, the system was equilibrated under constant pressure (NPT, 1 bar) while maintaining the temperature at 300.0 K, to achieve proper system density and prepare a stable starting structure for production simulations.

The production phase consisted of a 1 ns MD simulation under NPT conditions without any restraints. The system was maintained at 300.0 K and 1 bar, and the trajectories were recorded for further analysis. Structural stability and dynamics were evaluated using CPPTRAJ (Roe and Cheatham, 2013), including analysis of root mean square deviation (RMSD), radius of gyration (Rg), solvent-accessible surface area (SASA), root mean square fluctuation (RMSF), and the number of hydrogen bonds. Results were visualized using Matplotlib to compare conformational differences in ligand binding between the wild-type and mutant proteins.

Furthermore, the binding free energy between protein and ligand was estimated using the MM/GBSA method (Genheden and Ryde, 2015). A total of 1,000 snapshots were extracted from the last 10 ns of a 100 ns MD trajectory. The van der Waals, electrostatic, polar, and non-polar solvation contributions were calculated, and total binding free energies were reported in kJ/mol.

### 2.19 Statistical analysis

Statistical analyses were performed using SPSS 21.0 software. Continuous data were analyzed by one-way ANOVA with LSD post-hoc test or Kruskal-Wallis test, as appropriate. A p-value <0.05 was considered statistically significant for all analyses.

## 3 Results

### 3.1 THSW decoction improves liver inflammation and fibrosis in mice

**FIGURE 1:**
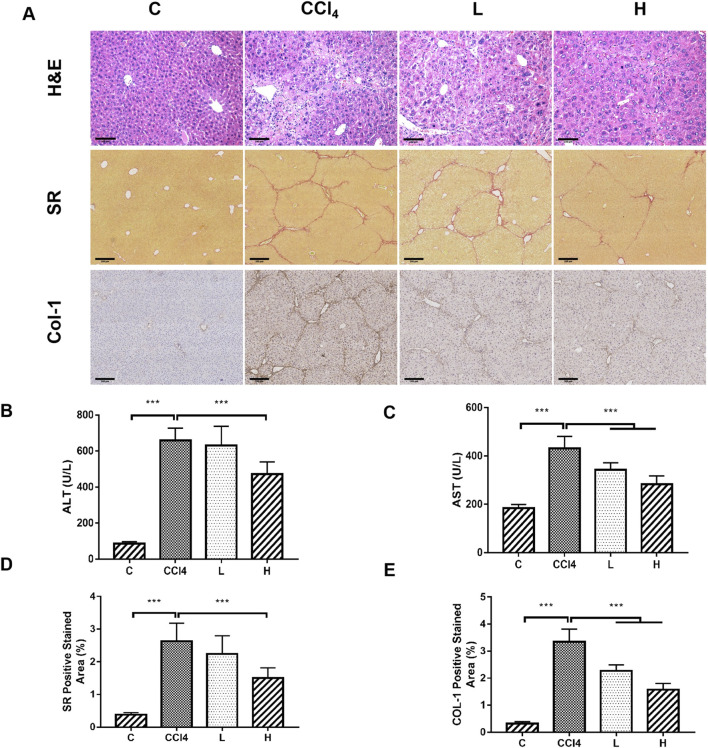
The ameliorative effect of THSW decoction on liver fibrosis in mice. **(A)**: Histopathological analysis of liver tissues, including H&E staining (magnification: ×200), Sirius Red (SR) staining (magnification: ×100), and collagen type I (Col-1) immunostaining (magnification: ×100). **(B)**: Serum alanine aminotransferase (ALT) levels. **(C)**: Serum aspartate aminotransferase (AST) levels. **(D)**: Semi-quantitative analysis of Sirius Red (SR) staining. **(E)**: Semi-quantitative analysis of Col-1 staining.

THSW Decoction significantly lowered serum ALT/AST levels, ameliorated hepatic collagen deposition (SR staining), and suppressed Col-1 expression in CCl_4_-induced mice, with the high-dose group exhibiting the most pronounced effects (Figure 1).

### 3.2 Identification of components absorbed into blood from THSW decoction

**FIGURE 2:**
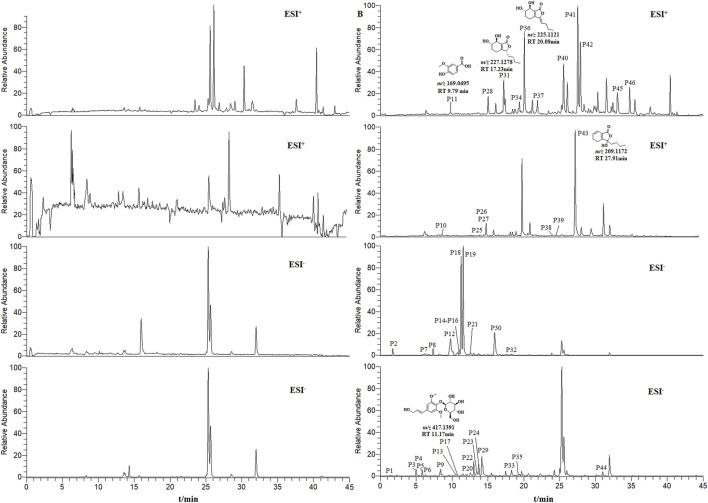
Extracted ion chromatograms (EICs) in positive and negative ionization modes from mouse serum samples collected before (left panels, blank serum) and after (right panels, post-administration serum) oral dosing of THSW Decoction. The displayed chromatograms correspond to the identified compounds: P11–Vanillic Acid; P17–Syringin; P31–Senkyunolide I; P36–Senkyunolide I; P43–Senkyunolide G.

THSW Decoction and serum samples from mice after administration of THSW Decoction were collected for data collection and qualitative analyses. A total of 231 chemical components were identified from the formula, of which 45 prototype chemical components were identified in the serum of mice administered THSW Decoction, including 14 phthalides, 8 each of phenolic acids and phenylpropanoids, 6 cyclic enol ether terpenoids, 4 alkaloids, 3 terpenoids, and 1 each of flavonoids and glycosides (Table 1; Figure 2).

**TABLE 1:** Prototype chemical components identified in the serum of mice administered THSW Decoction.

No.	RT/min	Ion mode	Measured mass/Da	Calculated mass/Da	Error/ppm	Molecular formula	Identification
P1	1.43	[M + HCOOH]^-^	421.1355	421.1341	1.447	C_16_H_24_O_10_	8-Epiloganic acid
P2	1.75	[M + HCOOH]^-^	407.1196	407.1184	1.668	C_15_H_22_O_10_	Catalpol
P3	5.00	[M + HCOOH]^-^	358.1147	358.1133	4.084	C_14_H_19_NO_7_	2-*β*-*D*-Glucopyranosyloxy-2-phenylacetic acid amide
P4	5.75	[M + HCOOH]^-^	731.2261	731.2241	2.014	C_27_H_42_O_20_	Rehmannioside D
P5	6.00	[M + HCOOH]^-^	569.1726	569.1712	1.363	C_21_H_32_O_15_	Melittoside
P6	6.29	[M-H]^-^	353.0879	353.0867	1.181	C_16_H_18_O_9_	Neochlorogenic acid
P7	6.73	[M-H]^-^	183.0290	183.0288	0.875	C_8_H_8_O_5_	Methyl gallate isomer
P8	7.37	[M + HCOOH]^-^	393.1406	393.1391	2.222	C_15_H_24_O_9_	Ajugol
P9	8.40	[M + HCOOH]^-^	389.1456	389.1442	1.377	C_16_H_24_O_8_	Mudanpioside F
P10	9.36	[M + H]^+^	195.0650	195.0652	−3.659	C_10_H_10_O_4_	Isoferulic acid
P11	9.79	[M + H]^+^	169.0493	169.0495	−0.205	C_8_H_8_O_4_	Vanillic acid
P12	9.80	[M-H]^-^	123.0438	123.0441	−0.306	C_7_H_8_O_2_	Guaiacol
P13	10.49	[M-H]^-^	353.0880	353.0867	1.241	C_16_H_18_O_9_	Cryptochlorogenic acid
P14	10.69	[M + HCOOH]^-^	502.1568	502.1555	1.254	C_20_H_27_NO_11_	Amygdalin
P15	10.78	[M-H]^-^	611.1619	611.1607	1.209	C_27_H_32_O_16_	Hydroxysafflor yellow A
P16	11.00	[M + HCOOH]^-^	315.1088	315.1074	2.563	C_13_H_18_O_6_	Benzyl-*β*-*D*-glucopyranoside
P17	11.17	[M + HCOOH]^-^	417.1404	417.1391	1.302	C_17_H_24_O_9_	Syringin
P18	11.27	[M + HCOOH]^-^	340.1038	340.1027	1.067	C_14_H_17_NO_6_	Prunasin
P19	11.55	[M + HCOOH]^-^	340.1039	340.1027	1.247	C_14_H_17_NO_6_	Prunasin isomer
P20	12.56	[M + HCOOH]^-^	525.1614	525.1603	1.113	C_23_H_28_O_11_	Albiflorin
P21	12.60	[M-H]^-^	163.0389	163.0390	−0.041	C_9_H_8_O_3_	P-Hydroxycinnamic acid
P22	12.69	[M + HCOOH]^-^	447.1511	447.1497	1.809	C_18_H_26_O_10_	Acetylcatalpol
P23	13.71	[M-H]^-^	367.1036	367.1024	1.191	C_17_H_20_O_9_	3-O-Feruloylquinic acid
P24	14.15	[M-H]^-^	449.1454	449.1442	1.310	C_22_H_26_O_10_	Prupersin A
P25	14.18	[M + H]^+^	179.0700	179.0703	−4.630	C_10_H_10_O_3_	Ferulaldehyde
P26	14.72	[M + H]^+^	195.0650	195.0652	−3.659	C_10_H_10_O_4_	Ferulic acid
P27	15.05	[M + H]^+^	225.1119	225.1121	−0.226	C_12_H_16_O_4_	Senkyunolide I isomer
P28	15.46	[M-H]^-^	239.0922	239.0914	0.968	C_12_H_16_O_5_	Senkyunolide S
P29	15.93	[M-H]^-^	137.0231	137.0233	−0.221	C_7_H_6_O_3_	Salicylic acid
P30	17.23	[M + H]^+^	227.1274	227.1278	−3.936	C_12_H_18_O_4_	Senkyunolide J
P31	18.28	[M-H]^-^	239.0923	239.0914	1.261	C_12_H_16_O_5_	Senkyunolide R
P32	18.41	[M-H]^-^	799.2672	799.2655	1.428	C_36_H_48_O_20_	Cistanoside A isomer
P33	19.36	[M + H]^+^	223.0962	223.0965	−3.648	C_12_H_14_O_4_	4,7-Dihydroxy-3-butylphthalide
P34	19.68	[M + HCOOH]^-^	435.2238	435.2225	1.673	C_19_H_34_O_8_	Rehmaglutin A/B
P35	20.08	[M + H]^+^	225.1118	225.1121	−3.838	C_12_H_16_O_4_	Senkyunolide I
P36	21.90	[M + H]^+^	223.0961	223.0965	−4.051	C_12_H_14_O_4_	Ethyl 4-hydroxy-3-methoxycinnamate isomer
P37	24.41	[M + H]^+^	189.0907	189.0910	−4.678	C_12_H_12_O_2_	E-Butylidenephthalide
P38	24.88	[M + H]^+^	207.1013	207.1016	−1.163	C_12_H_14_O_3_	Senkyunolide F
P39	25.55	[M + H]^+^	207.1013	207.1016	−1.356	C_12_H_14_O_3_	4-Hydroxy-3-butylphthalide
P40	27.55	[M + H]^+^	189.0907	189.0910	−4.678	C_12_H_12_O_2_	Z-Butylidenephthalide
P41	27.91	[M + H]^+^	191.1064	191.1067	−0.276	C_12_H_14_O_2_	3-Butylphthalide isomer
P42	27.91	[M + H]^+^	209.1170	209.1172	−1.056	C_12_H_16_O_3_	Senkyunolide G
P43	31.02	[M-H]^-^	203.0706	203.0703	−0.931	C_12_H_12_O_3_	Senkyunolide B
P44	33.05	[M + H]^+^	279.1585	279.1591	−4.027	C_16_H_22_O_4_	Senkyunolide M\Q
P45	34.78	[M + H]^+^	279.1587	279.1591	−1.417	C_16_H_22_O_4_	Dibutyl phthalate

### 3.3 Target screening for anti-hepatic fibrosis

**FIGURE 3:**
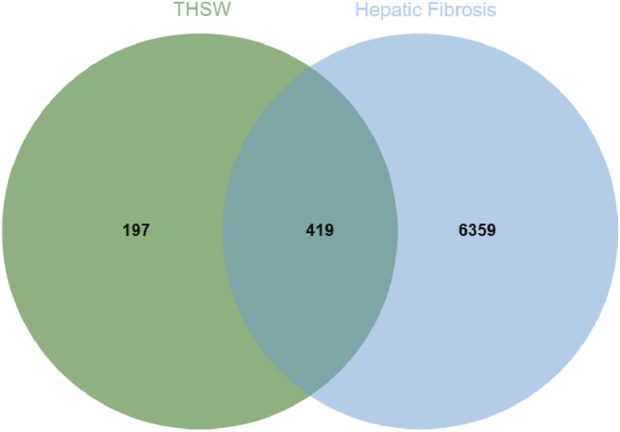
Potential anti-hepatic fibrosis targets of THSW Decoction.

To explore the potential targets of blood-entering components in THSW Decoction, target prediction analyses were performed using the SEA and TCMSP databases. First, the SMILES structures of the identified blood-entering compounds (retrieved from PubChem) were input into the SEA database. Based on molecular structure similarity, a total of 561 candidate protein targets were predicted. Subsequently, potential targets were also retrieved from the TCMSP database under the criteria outlined in Section 2.11, yielding 221 targets. After integrating the results from both databases and removing duplicates and invalid entries, a total of 616 unique potential targets were identified (Supplementary Table S1). Meanwhile, 102, 6,512, 21, 63, 31, and 1,216 targets were obtained from Drugbank, GeneCards, TTD, OMIM, PharmGkb, and DisGeNET databases, respectively. A total of 6,778 targets were obtained after deleting duplicates (Supplementary Table S2). The 419 targets duplicated between disease targets and components targets were considered potential targets for THSW Decoction against hepatic fibrosis (Figure 3; Supplementary Table S2).

### 3.4 PPI network and core targets

**FIGURE 4:**
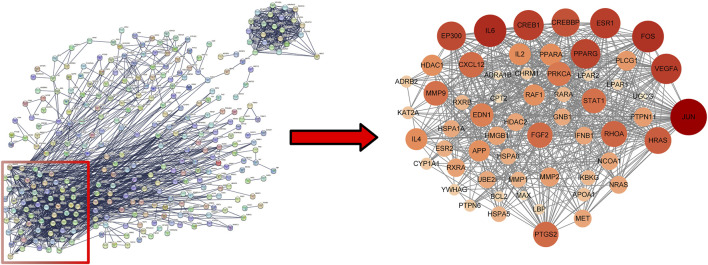
PPI network of core anti-hepatic fibrosis targets of components migrating to blood of THSW decoction.

Uploaded 419 component-disease targets to the STRING database and set a minimum interaction score of 0.9. A total of 4,529 edges, 419 nodes, and 59 core targets were obtained (Figure 4; Table 2; Supplementary Table S3). Cytoscape software was used to build the PPI network and assessed the degree value of each node. The node size and color brightness reflected the degree value size. These proteins are known to play a crucial role in the PPI network, which are the key targets for the active components of THSW Decoction to regulate liver fibrosis.

**TABLE 2:** Core anti-hepatic fibrosis targets of components migrating to blood of THSW decoction.

Target	Degree	Target	Degree	Target	Degree
EP300	48	RHOA	17	CHRM1	9
CREBBP	37	FOS	16	PTGS2	9
JUN	31	STAT1	16	IL4	9
HDAC1	29	APOA1	14	MAX	9
RXRA	26	HSPA8	14	PTPN6	9
NCOA1	25	PLCG1	14	MMP2	9
UBE2I	24	APP	13	ADRA1B	8
ESR1	23	NRAS	13	ADRB2	8
GNB1	22	IL2	13	YWHAG	8
HRAS	22	RXRB	12	KAT2A	8
PTPN11	20	RARA	12	FGF2	8
IL6	20	HSPA5	11	UGCG	7
EDN1	19	HDAC2	11	HSPA1A	7
VEGFA	19	MMP9	11	LBP	7
CXCL12	18	BCL2	10	MET	7
CYP1A1	18	RAF1	10	CPT2	6
PPARA	18	IFNB1	10	ESR2	6
CREB1	18	HMGB1	10	LPAR2	6
PRKCA	18	MMP1	10	-	-
PPARG	18	IKBKG	10	-	-

### 3.5 GO and KEGG enrichment

**FIGURE 5:**
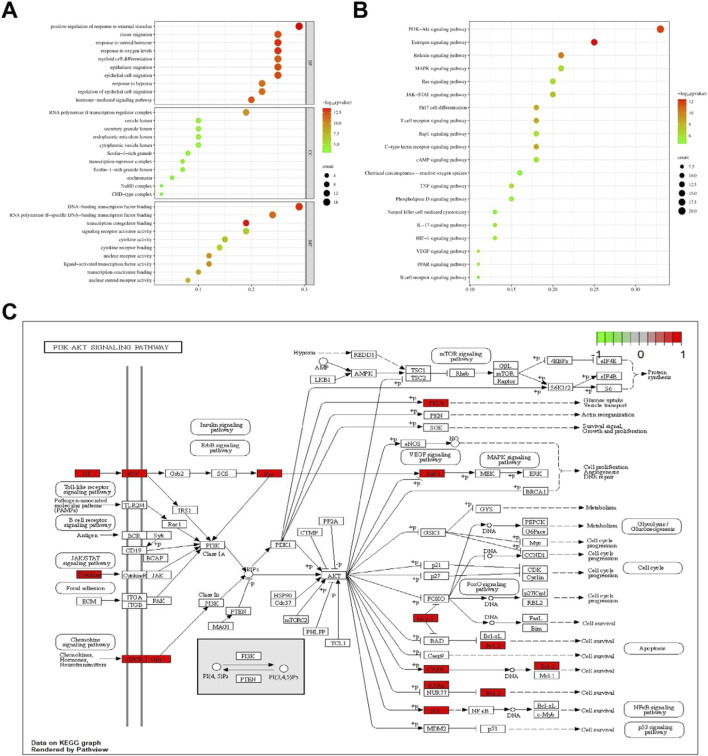
Enrichment analysis of core targets. **(A)**, GO enrichment analysis. The size and color of the bubbles represent the number of targets enriched and the corrected *P* value, respectively. **(B)**, KEGG signaling pathway enrichment analysis. **(C)**, Key targets of PI3K/AKT signaling cascade. Targets in red color represents potential targets of THSW Decoction.

GO enrichment analysis and KEGG pathway enrichment analysis were conducted on 59 core targets to further elucidate the mechanism of anti-hepatic fibrosis effect of THSW Decoction (Supplementary Table S4). A total of 1,518 BPs, 18 CCs, and 89 MFs were obtained from the GO functional annotations. BP mainly includes positive regulation of response to external stimulus, response to oxygen levels, epithelial cell migration, hormone-mediated signaling pathway, etc. CC mainly includes RNA polymerase II transcription regulator complex, transcriptional repressor complex, endoplasmic reticulum lumen, and vesicle lumen. MF mainly focuses on transcription coregulator binding, DNA-binding transcription factor binding, nuclear receptor activity, ligand-activated transcription factor activity (Figure 5A). The KEGG analysis indicated that the PI3K-Akt, estrogen, relaxin, and MAPK signaling pathways are likely the principal mechanisms through which THSW Decoction exerts its therapeutic effects on hepatic fibrosis (Figure 5B). Furthermore, the mechanism by which THSW Decoction regulates the PI3K-Akt signaling cascade in the context of anti-hepatic fibrosis was elucidated through visualization (Figure 5C).

### 3.6 Non-targeted metabolomics

#### 3.6.1 Multivariate statistical analysis

**FIGURE 6:**
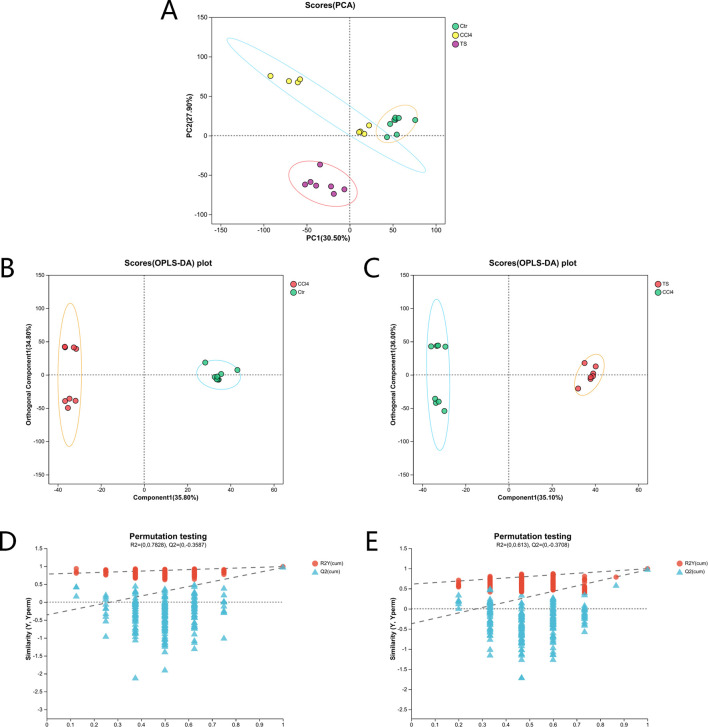
PCA and OPLS-DA analysis. **(A)** the PCA score plot. **(B)** the OPLS-DA score plot (normal group vs. model group). **(C)** the OPLS-DA score plot (model group vs. THSW group). **(D)** the permutation test plot of the OPLS-DA model (normal group vs. model group). **(E)** the permutation test plot of the OPLS-DA model (model group vs. THSW group).

The results of 3.1 showed that the high-dose group of THSW Decoction had the most significant curative effect in regulating liver fibrosis. Therefore, in this study, the liver tissues of mice in the high-dose group were selected as samples for non-targeted metabolomics. Principal component analysis (PCA) and orthogonal partial least squares discriminant analysis (OPLS-DA) were used for pattern recognition of the metabolic profile data of liver tissues, and sample distribution diagrams were obtained to reflect the similarities and differences among samples. The PCA results showed that there was an overlapping part between the normal group and the model group, while the THSW group was obviously separated from the model group. After pairwise comparison of the metabolites in the three groups of liver tissue samples by OPLS-DA, it was found that the discrimination degree among each group and the results of the permutation test were good (Figure 6).

#### 3.6.2 Differential Metabolite Mining and Metabolic Pathway Analysis of Liver Tissue Samples

**FIGURE 7:**
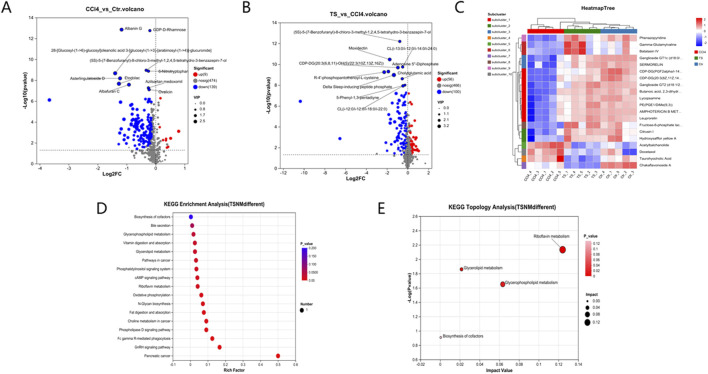
Differential Metabolite Mining and Metabolic Pathway Analysis of Liver Tissue Samples. **(A,B)**, the volcano plot analyses of differential metabolites. **(C)**, the cluster heatmap of biomarkers. **(D)**, Kegg pathway analysis. **(E)**, Pathway topological analysis.

Differential metabolites among the three groups of liver tissue samples were mined through the t-test. With the screening criteria of VIP >1 and P < 0.05, the volcano plot showed the expression trends and quantities of differential metabolites in the comparisons between groups. Compared with the normal group, 148 metabolites in the mice of the model group changed, among which the levels of 9 metabolites were upregulated and those of 139 metabolites were downregulated. Compared with the model group, the levels of 56 metabolites in the THSW Decoction group were upregulated and those of 100 metabolites were downregulated (Figure 7). To clarify how THSW Decoction exerts its therapeutic effect by influencing the levels of metabolites, the common differential metabolites obtained after pairwise comparisons of the metabolites among the three groups were selected as the main biomarkers (Table 3). The screened biomarkers were analyzed by cluster heatmap, and the results indicated that the normal group, the model group, and the THSW Decoction group clustered into different categories respectively, and the change trends of the normal group and the THSW Decoction group were basically consistent, which were opposite to those of the model group (Figure 7), suggesting that THSW Decoction may exert its pharmacological effects by regulating the biomarkers related to liver fibrosis.

**TABLE 3:** Biomarkers in liver tissue samples.

Metabolites	Trend of change		Ion mode	m/z
	CCL4 vs. Ctr	TS vs. CCL4		
Ganglioside GT1c (d18:0/12:0)	down	up	pos	535.14
Dtgammae	down	up	pos	207.06
CDP-DG (PGF2alpha/i-14:0)	down	up	pos	693.17
Mdz-glucuronide	down	up	pos	469.76
Integerrimine	down	down	pos	745.43
N,N′-Diacetylchitobiosyldiphosphodolichol	down	down	pos	1,041.52
1-tetradecanoyl-sn-glycero-3-phosphate	down	up	pos	1,121.54
Flavin mononucleotide	down	down	pos	682.67
(2s)-1-[1-(4-Phenylbutanoyl)-L-Prolyl]pyrrolidine-2-Carbonitrile	down	up	pos	1,119.62
Taurohyocholic Acid	up	down	pos	1,191.61
Lycopsamine	down	up	pos	1,316.70
CDP-DG (20:3 (8Z,11Z,14Z)-2OH(5,6)/22:6 (4Z,7Z,10Z,13Z,16Z,19Z))	down	up	pos	1,231.81
PI(5-iso PGF2VI/16:2 (9Z,12Z))	down	down	pos	1,073.70
Phenazopyridine	down	up	pos	439.10
Ganglioside GT2 (d18:1/22:0)	down	up	pos	1,031.59
AMPHOTERICIN B METHYL ESTER	down	up	pos	310.20
Acutoside B	down	down	pos	689.36
(5S)-5-(7-Benzofuranyl)-8-chloro-3-methyl-1,2,4,5-tetrahydro-3-benzazepin-7-ol	down	down	pos	580.62
Chakaflavonoside A	down	down	pos	436.73
SERMORELIN	down	up	pos	411.22
Fructose-6-phosphate lactate	down	up	pos	421.07
Leuprorelin	down	up	pos	310.14
PA (15:0/14:1 (9Z))	up	down	pos	358.16
Acetylbalchanolide	up	down	pos	556.15
Citrusin I	down	up	pos	327.01
PE (PGE1/DiMe(9,3))	down	up	pos	214.11
Estra-1,3,5 (10)-triene-3,17-diol, 16-iodo-, (16alpha,17beta)-	down	down	pos	262.14
Hydroxysafflor yellow A	down	up	pos	378.16
Brevetoxin B4b	up	down	pos	317.20
Butanoic acid, 2,3-dihydroxypropyl ester	down	up	pos	745.23
Docetaxol	up	down	pos	307.09
Gamma-Glutamylvaline	down	up	pos	347.20
Cholylglutamine	down	down	pos	128.02
Diethyl cyanophosphonate	up	down	neg	788.32
Batatasin IV	down	up	neg	857.45

Note: pos, positive ion mode; neg, negative ion mode.

Subsequently, KEGG metabolic pathway analysis and topological analysis (used to identify the principal metabolic pathways associated with the target metabolite set) were performed on the biomarkers listed in Table 3, as illustrated in Figure 7. The results demonstrated that the biomarkers were enriched in key pathways including cAMP, phospholipase D, and GnRH signaling pathways. Topological analysis further identified riboflavin metabolism as the most significant pathway.

### 3.7 Construction of the “drug-component-target-metabolite” network

**FIGURE 8:**
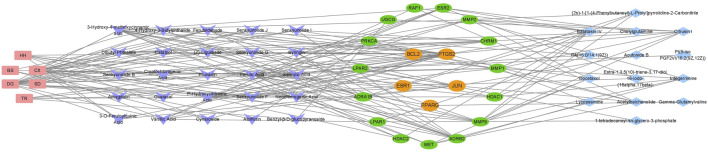
The “Drug-Component-Target-Metabolite” Network. Red nodes represent traditional Chinese medicine compounds, purple nodes indicate blood-absorbed components, while green and yellow nodes denote intersecting targets (with yellow highlighting core targets). Blue nodes represent differential metabolites.

A total of 35 chemical structures of common differential metabolites were retrieved from the PubChem database. Using the SwissTargetPrediction platform, we predicted 592 potential targets (Supplementary Table S5). After intersecting these with the targets of blood-absorbed components and liver fibrosis-related targets, we identified 20 overlapping targets (Table 4). These 20 intersection targets were then uploaded to the STRING database with an interaction score threshold set at 0.4, yielding a network with 70 edges and 18 nodes. This network was subsequently imported into Cytoscape 3.8.0 for topological analysis. The top five targets identified were JUN, PTGS2, BCL2, ESR1, and PPARG. From the “Drug-Component-Target-Metabolite” network, the highest-ranking blood-absorbed components were ferulic acid, p-hydroxycinnamic acid, 3-hydroxy-4-methoxycinnamic acid, ferulaldehyde, and vanillic acid (Figure 8).

**TABLE 4:** Intersection targets of THSW decoction components in blood-liver fibrosis-differential metabolite pathways.

Target	Degree
JUN	14
PTGS2	13
BCL2	13
ESR1	13
PPARG	12
MMP9	12
MMP2	9
ESR2	7
PRKCA	7
MET	6
MMP1	6
HDAC1	6
HDAC2	5
RAF1	5
LPAR1	4
ADRB2	3
LPAR2	3
CHRM1	2

### 3.8 Molecular docking

**FIGURE 9:**
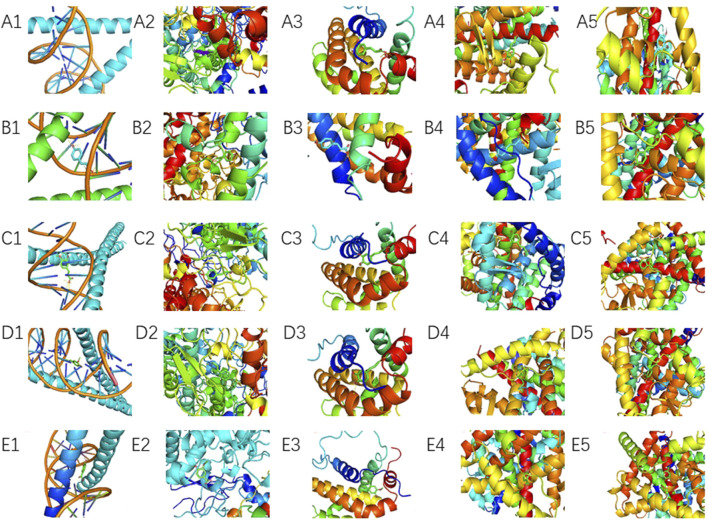
Molecular Docking Verification of the Core Components Entering the Blood of THSW Decoction and the Core Targets. **(A1–5)**, ferulic acid with JUN, PTGS2, BCL2, ESR1, PPARG. **(B1–5)**, p-hydroxycinnamic acid with JUN, PTGS2, BCL2, ESR1, PPARG. **(C1–5)**, 3-hydroxy-4-methoxycinnamic acid with JUN, PTGS2, BCL2, ESR1, PPARG. **(D1–5)**, ferulaldehyde with JUN, PTGS2, BCL2, ESR1, PPARG. **(E1–5)**, vanillic acid with JUN, PTGS2, BCL2, ESR1, PPARG.

Molecular docking was performed between the top five target genes (JUN, PTGS2, BCL2, ESR1, and PPARG) and the top five core blood-entering components (ferulic acid, p-hydroxycinnamic acid, 3-hydroxy-4-methoxycinnamic acid, ferulaldehyde, and vanillic acid), and the results were visualized (Table 5; Figure 9) to evaluate the binding affinities between target proteins and ligand molecules. Generally, a binding energy less than–5.0 kcal mol^-1^ indicates a significant interaction between the molecules. The results demonstrated that the core blood-entering components of THSW Decoction exhibited favorable binding affinities with the core targets.

**TABLE 5:** Binding energy of core components migrating to blood with core target.

Components migrating to blood of THSW decoction	Binding energy				
	JUN	PTGS2	BCL2	ESR1	PPARG
ferulic acid	−6.6	−6.5	−6.0	−6.5	−6.6
p-hydroxycinnamic acid	−5.9	−6.6	−5.6	−6.3	−6.5
3-hydroxy-4-methoxycinnamic acid	−6.8	−6.6	−5.9	−6.2	−6.8
ferulaldehyde	−6.5	−6.2	−5.9	−5.7	−6.0
vanillic acid	−6.6	−6	−5.6	−6.4	−5.9

Note: Typically, binding energy < −5.0 kcal·mol-1, indicate significant binding capacity between molecules. The smaller the value, the stronger the binding capacity.

### 3.9 Molecular dynamics simulation

To comprehensively evaluate the binding capacity of 3-hydroxy-4-methoxycinnamic acid with PPARG and compare it with the original ligand, a systematic analysis was conducted based on hydrogen bonding interactions, conformational stability (RMSD, RMSF, Rg), surface properties (SASA), and binding free energy (MM/GBSA).

#### 3.9.1 Hydrogen Bond Analysis

**FIGURE 10:**
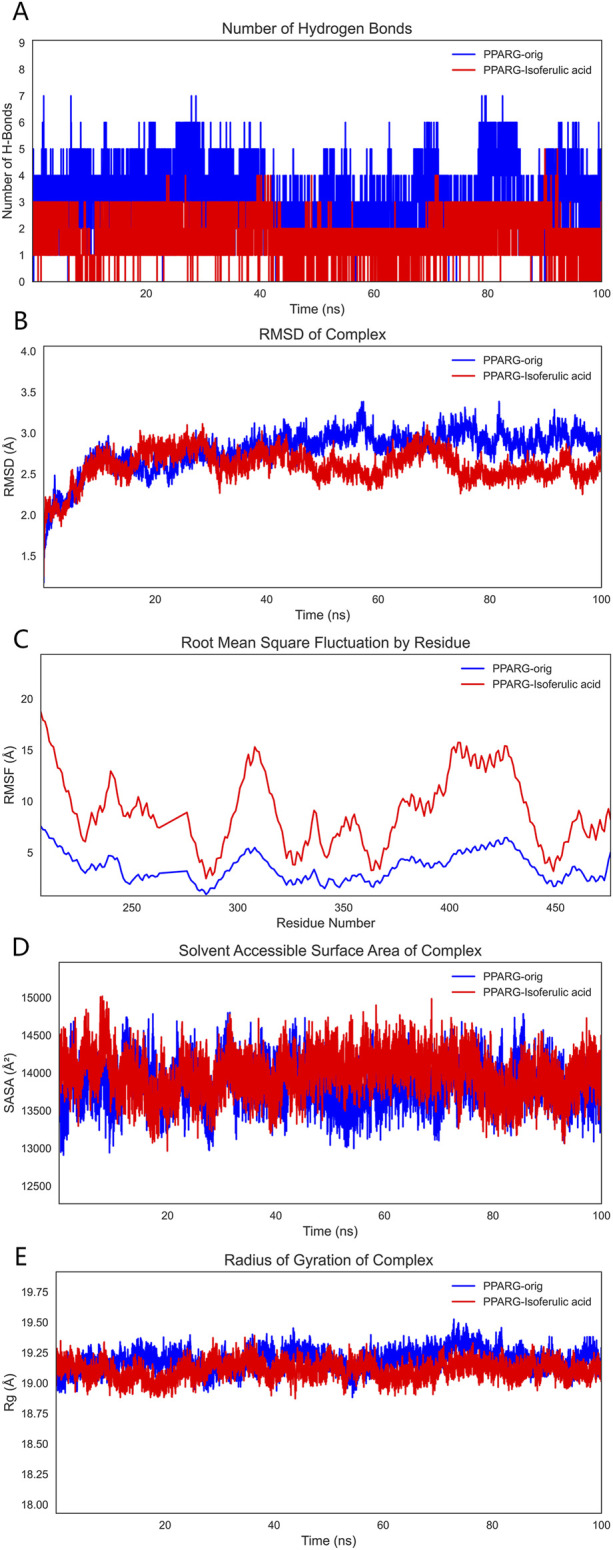
Molecular dynamics simulation results. Note: **(A)**, Hydrogen bond analysis; **(B)**, RMSD analysis; **(C)**, RMSF analysis; **(D)**, SASA analysis; **(E)**, Radius of gyration (Rg) analysis.

Hydrogen bonding, as a crucial non-covalent interaction, significantly stabilizes protein-ligand complexes through directional electrostatic attractions. During the 100 ns molecular dynamics simulation, the number of hydrogen bonds formed between the ligands and PPARG was monitored. The PPARG–3-hydroxy-4-methoxycinnamic acid complex consistently maintained 2–3 hydrogen bonds with minimal fluctuation, indicating relatively stable hydrophilic interactions. In comparison, the PPARG–original ligand complex formed 4–5 hydrogen bonds, which may be attributed to the presence of more polar groups in the original ligand. These findings suggest that both ligands can form stable hydrogen bond networks at the binding site (Figure 10A).

#### 3.9.2 RMSD analysis

RMSD (Root Mean Square Deviation) was employed to assess the overall conformational stability of the protein–ligand complexes. The RMSD trajectories indicated that both complexes maintained good stability throughout the simulation, with fluctuations remaining within 2 Å and no significant drift observed. This suggests that both systems were thermodynamically stable during the simulation period and were thus suitable for further binding energy analysis (Figure 10B).

#### 3.9.3 RMSF analysis

RMSF (Root Mean Square Fluctuation) was used to evaluate the local flexibility of PPARG residues. The binding site residues in the PPARG–3-hydroxy-4-methoxycinnamic acid complex displayed moderate fluctuations around 10 Å, whereas the corresponding residues in the PPARG–original ligand complex exhibited lower RMSF values, indicating a stronger local stabilizing effect by the original ligand. Low flexibility at the binding site is typically associated with tighter molecular recognition, suggesting the original ligand may better stabilize the functional conformation of the protein (Figure 10C).

#### 3.9.4 SASA analysis

SASA (Solvent Accessible Surface Area) reflects the degree of exposure of the protein surface to the solvent and is a key parameter for assessing conformational changes and hydrophilic/hydrophobic surface exposure. The SASA curve of the PPARG–3-hydroxy-4-methoxycinnamic acid complex showed a slight upward trend during the simulation, likely due to increased exposure of polar groups. In contrast, the PPARG–original ligand complex displayed more stable SASA values, suggesting a more compact structure with better shielding of hydrophobic regions. Overall, both complexes exhibited stable SASA behavior, although 3-hydroxy-4-methoxycinnamic acid may slightly increase solvent exposure, potentially affecting solubility or bioactivity (Figure 10D).

#### 3.9.5 Rg analysis

The radius of gyration (Rg) was analyzed to reflect the compactness of the protein structure. Both PPARG–ligand complexes demonstrated stable Rg values with minimal fluctuation, indicating a tight and well-folded protein conformation that supports functional stability (Figure 10E).

#### 3.9.6 MM/GBSA binding free energy analysis

To quantitatively evaluate ligand–PPARG binding affinities, MM/GBSA calculations were performed using 100 frames extracted from the last 1 ns of the 100 ns MD trajectory. The binding free energy (ΔG_binding) and its components are summarized, respectively. As shown in Table 6, the PPARG–original ligand complex exhibited a binding free energy of −166.89 ± 17.86 kJ/mol, primarily driven by van der Waals interactions (−212.34 kJ/mol) and electrostatic contributions (−102.39 kJ/mol). These results indicate a strong binding affinity, largely governed by favorable non-covalent interactions. In contrast, Table 7 presents the energy components for the PPARG–3-hydroxy-4-methoxycinnamic acid complex. This complex displayed a higher ΔG_binding of −93.68 ± 9.12 kJ/mol. Notably, the electrostatic interaction term was positive (+18.40 kJ/mol), suggesting relatively unfavorable polar interactions compared to the original ligand. The reduced van der Waals contribution (−97.53 kJ/mol) also reflects a weaker overall binding affinity.

**TABLE 6:** Free energy distribution of PPARG with the original ligand.

Energy type	Compound (kJ/mol)
E_VDW: van der Waals energy	−212.3434 ± 13.2378
E_ELE: electrostatic energy	−102.3946 ± 47.3194
E_GB: electrostatic contribution to solvation	180.3614 ± 35.3326
E_SA: non-polar contribution to solvation	−32.5047 ± 0.8678

**TABLE 7:** Free energy distribution of PPARG with 3-hydroxy-4-methoxycinnamic acid.

Energy type	Compound (kJ/mol)
E_VDW: van der Waals energy	−97.5269 ± 8.8579
E_ELE: electrostatic energy	18.3970 ± 24.7714
E_GB: electrostatic contribution to solvation	2.0355 ± 21.7815
E_SA: non-polar contribution to solvation	−16.5820 ± 0.6799
ΔG_binding: binding free energy	−93.6764 ± 9.1224

### 3.10 *In Vivo* target validation

**FIGURE 11:**
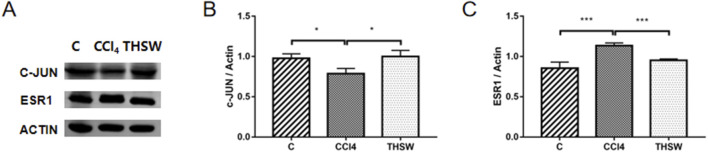
Effects of THSW Decoction on the Core Liver Targets (JUN, ESR1) in Mice with Liver Fibrosis. **(A)**, Protein expressions of c-jun and ESR1 in the liver. **(B,C)**, Semi-quantification of the protein expressions of c-jun and ESR1 in the liver. *P < 0.05, ***P < 0.001.

Finally, animal experiments were conducted to verify the anti-liver fibrosis mechanism of THSW Decoction. The results further confirmed the effect of the high dose of THSW Decoction significantly increased JUN expression in the liver tissues of mice with liver fibrosis and decreased ESR1 expression (Figure 11).

## 4 Discussion

Hepatic fibrosis is characterized by excessive deposition of collagen and other extracellular matrix (ECM) components such as fibronectin and proteoglycans. Various factors - including viral infections, alcohol consumption, obesity, and autoimmune disorders-can trigger hepatic fibrosis. These agents act through multiple pathways, including promoting liver inflammation, oxidative stress, and apoptosis (Altamirano-Barrera et al., 2017). While these pathogenic factors are well-established, therapeutic options remain limited. Interestingly, traditional Chinese medicine formulations such as THSW Decoction, have shown promise in fibrosis treatment (Xi et al., 2016), but its pharmacologically active components and precise mechanisms of action remain incompletely understood.

In this study, THSW Decoction’s efficacy in improving hepatic fibrosis was first evaluated by constructing a murine model of hepatic fibrosis induced by carbon tetrachloride. The results demonstrated that THSW Decoction significantly reduced serum alanine aminotransferase (ALT) and aspartate aminotransferase (AST) levels in CCl4-induced hepatic fibrosis mice, ameliorated collagen deposition in liver tissues, and decreased Col-1 expression, indicating its effectiveness in alleviating liver inflammation and fibrosis. To explore its potential active components and mechanism of action, this study further employed UHPLC-Q-Exactive Orbitrap HRMS technology to analyze and identify the main prototype chemical components of THSW Decoction in the blood. A total of 45 blood components of THSW Decoction were detected. Database searches yielded 616 drug targets corresponding to 43 blood components, among which 419 were identified as potential anti-hepatic fibrosis targets. Through GO and KEGG enrichment analyses, it was revealed that the mechanism of THSW Decoction in treating liver fibrosis involves multiple aspects. In the GO enrichment analysis, the top-ranked functions include response to oxygen levels, nuclear receptor activity, ligand-activated transcription factor activity, etc. Cells need continuous oxygen supply to power energy production through mitochondrial respiration. Hypoxia can activate hypoxia - inducible factors (HIFs), which then affect the respiratory chain function and cause an increase in reactive oxygen species (ROS). Under persistent hypoxia, HIFs coordinate interactions among hepatocyte populations, promote pro - angiogenic mediator release, and drive inflammatory and fibrotic responses (Foglia et al., 2021). As ligand-regulated transcription factors, nuclear receptors control inflammation. They also mediate hepatic stellate cell (HSC) activation (Tran et al., 2018; Rudraiah et al., 2016). Building on these molecular mechanisms, our KEGG enrichment analysis reveals that THSW Decoction treats liver fibrosis mainly involves the Phosphatidylinositol-3-Kinase/Protein Kinase B(PI3K-Akt) pathway, along with estrogen, relaxin, and Mitogen-Activated Protein Kinase (MAPK) signaling pathways. The PI3K-Akt pathway promotes liver fibrosis through three key mechanisms: stimulating ECM production, activating hepatic stellate cells (HSCs), and inducing hepatic sinusoidal capillarization (Wang M. et al., 2022; Wang et al., 2019). Estrogen inhibits the activity of Kupffer cells and reduces the production of ROS via estrogen receptor α (ERα/ESR1), exerting a protective effect on various liver diseases (Kovats, 2015; Xu et al., 2022). Relaxin signals via its cognate receptor RXFP1, a G protein - coupled receptor, to: (1) inhibit collagen synthesis/deposition, (2) suppress HSC activation, and (3) enhance ECM degradation while stimulating angiogenesis (Samuel et al., 2017; Kaftanovskaya et al., 2019). Three MAPK subfamilies, namely, p38 MAPK, JNK, and ERK1/2-collectively drive HSCs activation and fibrogenesis (Jin et al., 2022; Zhang et al., 2021).

Building on network pharmacology predictions, we employed non-targeted metabolomics using a Synapt G2-Si QTOF HRMS system to correlate predicted targets and pathways with actual metabolic changes observed *in vivo*, systematically elucidating the overall regulatory mechanisms of traditional Chinese medicine treatment. Non-targeted metabolomics revealed 148 and 156 differential metabolites when comparing model vs. normal groups and THSW Decoction vs. model groups, respectively. Among them, 35 common differential metabolites were identified, and these were enriched in signaling pathways such as cAMP, Phospholipase D, and gonadotropin-releasing hormone (GnRH). The cAMP signaling pathway regulates liver metabolism through gene transcription and kinase activity modulation. As demonstrated previously (Cheng et al., 2008; Wahlang et al., 2018), cAMP regulates the expression of protein kinase A (PKA) and cAMP sensor family proteins (EPAC), inhibits the activation and proliferation of HSCs, regulates the synthesis of the ECM, and suppresses the inflammatory response to alleviate the degree of liver fibrosis. These findings experimentally validate the PI3K-Akt pathway predictions from our network pharmacology analysis. Based on different cellular localizations and functions, Phospholipase D can be divided into two subtypes: PLD1 and PLD2. Studies demonstrate that the expression level of PLD1 typically increases with HSCs activation, while PLD2 remains relatively unchanged (Chang et al., 2020). Targeting PLD1 can inhibit liver fibrosis. Phosphatidic acid (PA), a key PLD pathway metabolite, activates the extracellular signal-regulated kinase (ERK) pathway. This regulates DNA synthesis while influencing HSCs activation and proliferation (Benitez-Rajal et al., 2006). PA also binds to peroxisome proliferator-activated receptor alpha (PPARα), modulating lipid metabolism (Yao et al., 2024). THSW Decoction downregulates the level of PA. This suggests THSW Decoction may ameliorate liver fibrosis through a metabolism-transcription coupling mechanism. Research indicates that GnRH binds to HSCs surface receptors and regulates miR-200b expression, potentially participating in HSCs activation through metabolic reprogramming (Kyritsi et al., 2017; Xie et al., 2023).

This study investigated how THSW Decoction combats liver fibrosis by combining network pharmacology and metabolomics approaches. We identified 20 overlapping targets connecting blood - absorbed components, metabolites, and liver fibrosis. PPI analysis revealed five key targets:JUN, PTGS2, BCL2, ESR1, and PPARG, indicating THSW Decoction’s multi-target anti-fibrotic mechanism. As a key downstream target of the mitogen-activated protein kinase (MAPK) pathway, JUN phosphorylation promotes HSCs proliferation and the release of inflammatory factors. THSW Decoction significantly downregulated JUN expression, potentially inhibiting HSC activation through MAPK-JUN cascade blockade (Kluwe et al., 2010; Win et al., 2018). PTGS2 drives liver fibrosis via three key mechanisms: under the induction of inflammatory factors, PTGS2 catalyzes PGE2 production, exacerbating liver inflammation (Takai et al., 2013; Rodriguez-Barbero et al., 2006). Additionally, it participates in HSCs activation by inhibiting autophagy-related proteins and upregulating α-SMA expression (Yang et al., 2020). The anti-apoptotic protein BCL2 regulates cytochrome C release from mitochondria by binding to BCL2-associated agonist of cell death (BAD), thus modulating cell apoptosis. THSW Decoction demonstrates anti-fibrotic effects by regulating BCL-2 expression to promote HSCs apoptosis, consistent with previous research (Meng et al., 2018). The functions of ESR1 were previously discussed. It inhibits liver fibrosis in both basic and clinical research (Xu et al., 2004; Cengiz et al., 2014). PPAR (peroxisome proliferator-activated receptor) serves as a potential liver-fibrosis treatment target. It alleviates fibrosis by suppressing HSCs activation, modulating inflammation/oxidative stress, improving lipid metabolism, and regulating immune responses. Clinical studies have validated PPAR agonists’ efficacy (Li et al., 2021; Liu et al., 2020).

Through network pharmacology analysis establishing a “drug-component-target-metabolite” interaction map, five components (ferulic acid, p-hydroxycinnamic acid, 3-hydroxy-4-methoxycinnamic acid, ferulaldehyde, and vanillic acid) showed high degree values among detected blood-entering components, while differential metabolites such as Batatasin IV, Citrusin I, Acetylbalchanolide, and Integerrimine also exhibited high degree values, indicating that these compounds and metabolites may serve as key bioactive substances in the therapeutic effects of THSW Decoction against liver fibrosis. ferulic acid, a derivative of cinnamic acid, has been demonstrated to possess anti-fibrotic properties in various liver fibrosis models (Shi et al., 2023). It may alleviate the intrahepatic inflammatory microenvironment by targeting PTGS2 to inhibit COX-2 expression (Gollapalli et al., 2024), and may also regulate fibrosis-related gene expression via PPARG (Cui et al., 2022; Ni et al., 2021). p-hydroxycinnamic acid, a natural phenolic compound, was reported to suppress the TLR4/NF-κB signaling pathway, reduce IL-1β precursor expression, and attenuate NLRP3 inflammasome activation in a mouse model of high-fat, high-sucrose diet-induced liver fibrosis, thereby inhibiting HSCs activation (Truong et al., 2023). 3-hydroxy-4-methoxycinnamic acid (Su et al., 2015) and ferulaldehyde (Radnai et al., 2009) also exhibit potent anti-inflammatory and antioxidant activities, although their specific roles in liver fibrosis require further investigation. In a CCl_4_-induced liver injury model, vanillic acid was found to significantly inhibit HSCs activation and collagen accumulation, demonstrating anti-fibrotic potential (Itoh et al., 2010), while also can regulate BCL2 expression to suppress apoptosis (Punvittayagul et al., 2021). Among the differential metabolites, Batatasin IV and Citrusin I were upregulated in the THSW Decoction group, suggesting that the THSW Decoction may promote the biosynthesis of metabolites with anti-inflammatory and antioxidant properties by modulating relevant pathways (Yang et al., 2009; Noh et al., 2015). Integerrimine, a pyrrolizidine alkaloid (PA) with known hepatotoxicity and diverse metabolic routes, was downregulated, indicating that THSW Decoction might suppress toxic PA bioactivation, helping to mitigate liver injury while maintaining anti-fibrotic efficacy (Wang Z. et al., 2022). Acetylbalchanolide, a sesquiterpene lactone, remains poorly studied and merits further investigation. Interestingly, beyond the top-ranked metabolites, PA (15:0/14:1 (9Z)) was enriched across multiple pathways and downregulated in the THSW Decoction group, potentially due to phospholipase D pathway inhibition. This suggests THSW Decoction may help restore phospholipid metabolic balance and regulate intercellular signal transduction (Sarkar and Chattopadhyay, 2021). Overall, the “drug–component–target–metabolite” network highlights that THSW Decoction may exert its anti-fibrotic effects through the synergistic action of multiple bioactive compounds. These compounds coordinately modulate key molecular targets involved in the pathogenesis of liver fibrosis, particularly pathways related to inflammation and oxidative stress. Consequently, THSW Decoction may influence critical metabolic pathways and associated metabolite levels, ultimately contributing to a multifaceted therapeutic intervention against liver fibrosis.

To validate the relationship between the main components of THSW Decoction and its core targets, molecular docking was used to predict their binding affinities. Ferulic acid, p-hydroxycinnamic acid, 3-hydroxy-4-methoxycinnamic acid, ferulaldehyde, and vanillic acid showed strong binding capacities with JUN, PTGS2, BCL2, ESR1, and PPARG. These findings suggest the primary bioactive components of THSW Decoction (ferulic acid, p-hydroxycinnamic acid, 3-hydroxy-4-methoxycinnamic acid, ferulaldehyde, and vanillic acid) may inhibit liver fibrosis by modulating JUN, PTGS2, BCL2, ESR1, and PPARG. This modulation could exert anti-inflammatory and anti-oxidative effects while regulating ECM production and HSCs activation. In the molecular dynamics simulations, the original ligand exhibited stronger binding affinity and a more stable complex structure. Nevertheless, 3-hydroxy-4-methoxycinnamic acid also maintained a stable conformation throughout the 100 ns simulation (RMSD <2 Å), established a consistent hydrogen bond network, and showed a favorable binding free energy (−93.68 kJ/mol), suggesting its ability to form a thermodynamically favorable complex with PPARG. Taken together, these results indicate that 3-hydroxy-4-methoxycinnamic acid, as a natural compound, demonstrates appreciable binding potential toward PPARG, providing theoretical support for its candidacy as a PPARG modulator. *In vivo* experiments showed that THSW Decoction significantly upregulated JUN expression and downregulated ESR1, which was consistent with the network prediction results. However, the direct interaction between these blood-entering components and their predicted targets requires verification, and this will be addressed in future work.

## Data Availability

The datasets presented in this study can be found in online repositories. The names of the repository/repositories and accession number(s) can be found in the article/Supplementary Material.
