# Supplementary material for: Non-targeted metabolomics and network pharmacology of Taohong Siwu Decoction in hepatic fibrosis mouse model using high resolution mass spectrometry
Source: Front Mol Biosci. 2025 Jun 30;12:1614341. doi: 10.3389/fmolb.2025.1614341 (PMC12256948; doi:10.3389/fmolb.2025.1614341)
Supplement: Supplementary file 1 [file DataSheet1.zip › Supplementary Files/Supplementary Materials.docx]

1、Non-targeted Metabolomics Analysis of Liver Tissue

1.1、Chromatographic and Mass Spectrometric Conditions

Serum samples (100 µL) were deproteinized by adding four volumes of acetonitrile:methanol (4:1, v/v), vortexed for 3 min, and incubated at 4°C for 30 min. After centrifugation at 13,000 rpm for 15 min, the supernatant was collected, evaporated to dryness under vacuum, and reconstituted in 50 µL of 50% acetonitrile in water. Quality control (QC) samples were prepared by pooling equal volumes of all individual samples and injected periodically throughout the analysis to assess system stability and reproducibility.

UPLC analysis was performed on an ACQUITY UPLC system (Waters, Milford, MA, USA) using an ACQUITY UPLC BEH C18 column (2.1 × 100 mm, 1.8 μm). The mobile phases consisted of 0.1% formic acid in water (solvent A) and 0.1% formic acid in acetonitrile (solvent B). The gradient program was as follows: 0–5 min, 2%–40% B; 5–17 min, 40%–98% B; 18–20 min, 100% B; and 20.1–21 min, 2% B. The flow rate was 0.3 mL/min, and the injection volume was 2 µL.

Mass spectrometric analysis was carried out using a Waters Synapt G2-Si QTOF mass spectrometer (Waters, Milford, MA, USA) equipped with an electrospray ionization (ESI) source operated in both positive and negative ion modes. The capillary voltage was set to 3.0 kV (ESI⁺) and 2.5 kV (ESI⁻), and data were acquired over the m/z range of 100–1000. Other MS parameters were optimized as recommended by the manufacturer.

To ensure mass accuracy, a two-level calibration strategy was employed. Full calibration was performed weekly or after instrument maintenance using sodium formate clusters (Waters QTOF Calibration Kit). Although the theoretical calibration coverage spans m/z 50–2000, the actual calibrated ion ranges were m/z 68.9957 ([HCOONa+H]⁺) to 1350.7810 ([15HCOONa+Na]⁺) in ESI⁺ mode, and m/z 112.9856 (CF₃COO⁻) to 1022.8239 ([8HCOONa+CF₃COO⁻]) in ESI⁻ mode, achieving a target mass error within ±2 ppm.

Daily mass correction was performed using Leucine Enkephalin (LE, 1 µg/mL in 50% acetonitrile with 0.1% formic acid) as a lock mass compound, with reference ions at m/z 556.2771 ([M+H]⁺) and 554.2615 ([M–H]⁻) for ESI⁺ and ESI⁻ modes, respectively. Lock mass calibration files were generated using the MS Calibration Tool in MassLynx software and applied throughout the run. All calibration procedures adhered to the manufacturer’s guidelines (Synapt G2-Si Mass Calibration Guide, Waters Corporation).

1.2、Detection Method

① Sample Pretreatment: A total of 100 μL of each sample was mixed with 400 μL of an extraction solution containing an internal standard (methanol:acetonitrile, 1:1, v/v; internal standard concentration: 2 μg/mL). The mixture was vortexed for 30 s and sonicated in an ice-water bath for 5 min. Samples were then incubated at −20°C for 1 h and centrifuged at 12,000 rpm for 15 min at 4°C. A 425 μL aliquot of the supernatant was transferred to an EP tube and evaporated to dryness in a vacuum concentrator.

②The dried metabolites were reconstituted in 100 μL of extraction solution (acetonitrile:water, 1:1, v/v), vortexed for 30 s, and sonicated for 10 min in an ice-water bath. The solution was centrifuged again at 12,000 rpm for 15 min at 4°C. Then, 60 μL of the supernatant was transferred to a 2 mL LC-MS vial for subsequent analysis.

1.3、Data Processing and Bioinformatics Analysis

Raw LC-MS data were imported into Progenesis QI software (Waters Corporation, Milford, MA, USA) for preprocessing, including baseline filtering, peak detection, integration, retention time correction, and peak alignment. A data matrix containing retention time, mass-to-charge ratio (m/z), and peak intensity was generated. The matrix was further processed by filtering, gap filling, normalization, and log transformation.Metabolite identification was performed by matching MS and MS/MS spectral data with public databases such as the Human Metabolome Database (HMDB, http://www.hmdb.ca/) and Metlin (https://metlin.scripps.edu/).Multivariate statistical analyses, including principal component analysis (PCA) and orthogonal partial least squares discriminant analysis (OPLS-DA), were conducted using the ropls package (version 1.6.2) in R. A seven-fold cross-validation approach was used to evaluate model stability.Differential metabolites were mapped to metabolic pathways using the KEGG database (https://www.kegg.jp/kegg/pathway.html). Pathway enrichment analysis was performed using the scipy.stats module in Python. Key pathways relevant to the intervention were identified based on topological analysis using the relative betweenness centrality method.

2、Detection of Blood Components of THSW Decoction

2.1、Chromatographic Conditions

UHPLC analysis for the prototype chemical components of THSW Decoction was performed using an Ultimate 3000 system (Dionex, USA), equipped with an online vacuum degasser, quaternary pump, autosampler, and a thermostatted column compartment, all controlled via Chromeleon 7.2 software. Chromatographic separation was achieved on a Waters Acquity UPLC BEH C18 column (2.1 × 100 mm, 1.7 μm). The column temperature was maintained at 40°C and the injection volume was 2 μL. The binary mobile phase consisted of solvent A (0.1% formic acid in water, v/v) and solvent B (methanol), with a flow rate of 0.3 mL/min. The gradient elution program was as follows: 0–1 min, 4% B; 1–4 min, 4–48% B; 4–24 min, 48–52% B; 24–26 min, 52–95% B; 26–28 min, 95% B; 28–28.01 min, 95–4% B; 28.01–30 min, 4% B. The total run time was 30 minutes.

2.2、Mass Spectrometry Conditions

Mass spectrometry analysis was carried out using a Q Exactive Orbitrap high-resolution mass spectrometer (Thermo Fisher Scientific, USA) coupled with the UHPLC system via a heated electrospray ionization (HESI) source. The instrument operated in both positive and negative ionization modes with a full MS scan range of m/z 100–1500. The following MS settings were applied: spray voltage, 3.5 kV (positive) and −2.5 kV (negative); capillary temperature, 325°C; sheath gas flow rate, 45 arbitrary units; auxiliary gas, 8 units; sweep gas, 0 units; S-lens RF level, 50 V; auxiliary gas heater temperature, 300°C. Data were acquired using Full MS/SIM and Full MS/data-dependent MS² (dd-MS²) scan modes, with a resolution of 70,000 FWHM for MS1 and 17,500 FWHM for MS2. Stepped normalized collision energies of 10, 20, and 40 eV were used for fragmentation. External mass calibration was performed daily using the manufacturer’s standard calibration solution to ensure mass accuracy in both ion modes.

Data acquisition and analysis were performed using Xcalibur 3.0 software. Molecular formulas were deduced based on precursor ions ([M+H]⁺, [M-H]⁻) and adduct ions ([M+Na]⁺, [M+HCOOH]⁻), with a mass error tolerance of ±10 ppm. Identification was achieved by comparing experimental data with reference standards, literature, and MS² fragments from the MassBank database.
